# Effect of carrier yeast RNAs in the detection of SARS-CoV-2 by RT-LAMP

**DOI:** 10.17912/micropub.biology.000979

**Published:** 2023-09-20

**Authors:** Samantha Morais-Armas, Sara Medina-Suárez, Félix Machín

**Affiliations:** 1 Unidad de Investigación, Hospital Universitario Nuestra Señora de Candelaria, Santa Cruz de Tenerife, Canary Islands, Spain; 2 Instituto de Tecnologías Biomédicas, Universidad de La Laguna, San Cristóbal de La Laguna, Canary Islands, Spain; 3 Facultad de Ciencias de la Salud, Universidad Fernando Pessoa Canarias, Las Palmas de Gran Canaria, Canary Islands, Spain

## Abstract

The COVID-19 pandemic caused by SARS-CoV-2 has underscored the need for rapid and accurate diagnostic methods. Reverse Transcription Loop-Mediated Isothermal Amplification (RT-LAMP) has emerged as a promising molecular tool in least developed countries due to its simplicity, speed, and sensitivity. Nevertheless, reliable SARS-CoV-2 detection can be challenged by the chain custody of the samples. In this context, carrier RNA can act as a preservative. In this study, we explored the potential of yeast total and transference RNA (tRNA) in the SARS-CoV-2 RT-LAMP. We have found that most optimal conditions are reached with 1 μg/μL tRNA in the RT-LAMP reaction.

**Figure 1. Characterization of yeast RNAs as additives in SARS-CoV-2 RT-LAMP:**
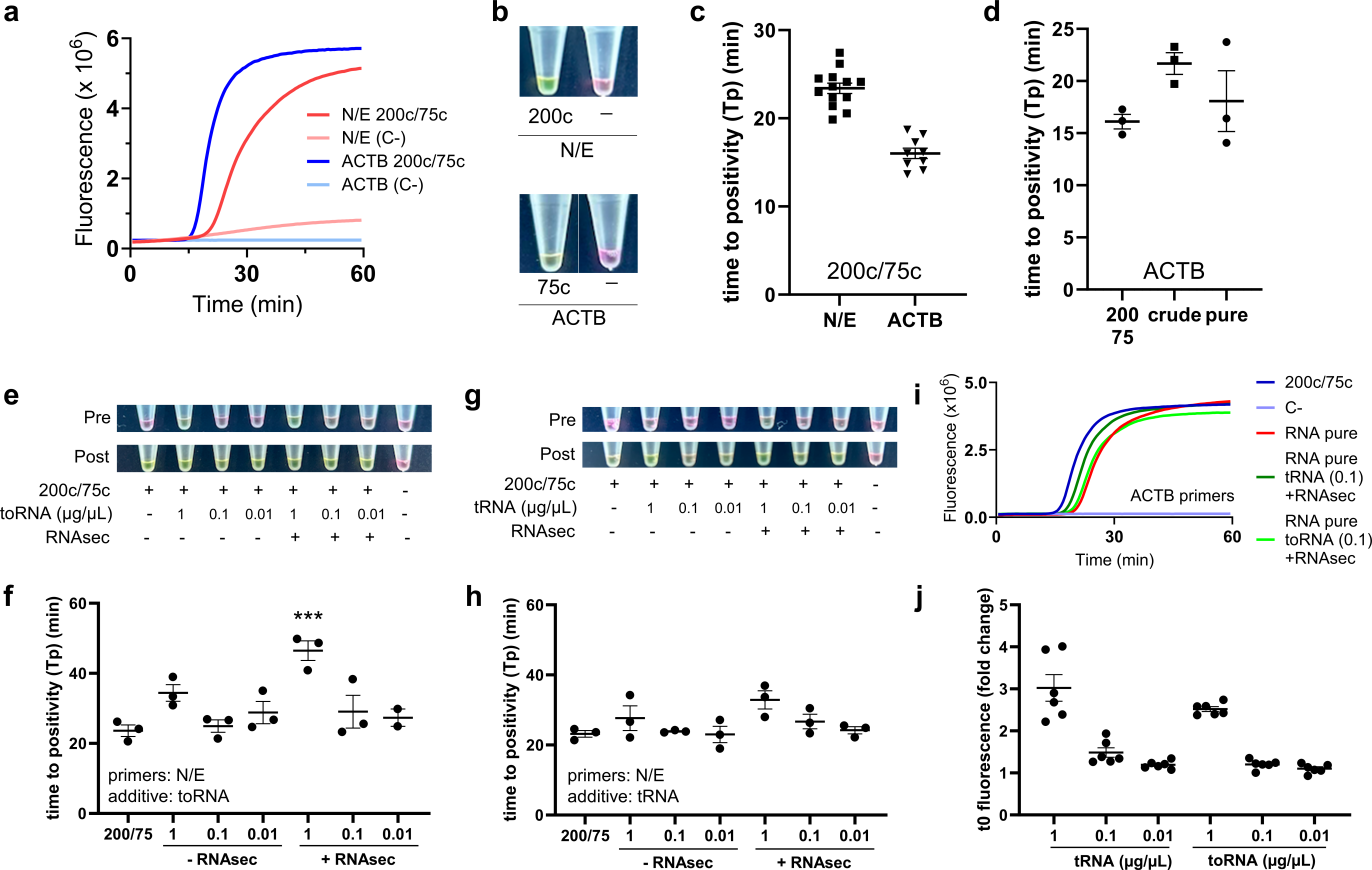
( **a** ) Representative amplification profile of the RT-qLAMP with the N/E and ACTB primers against the 200c/75c template. No template (C-) is also included as a control. ( **b** ) Representative colorimetric end point result after 60’ of the RT-qLAMP shown in (a). ( **c** ) Reproducibility of the amplification results shown in (a). ( **d** ) Comparison of amplification timing for ACTB between mid-nasal swabs (crude and pure extracted RNA) and the 200c/75c template. Differences were n.s. (one-way ANOVA). ( **e** ) Effect of yeast total RNA and RNAsecure in end-point colorimetric RT-qLAMP against SARS-CoV-2 RNA. Upper and lower pictures show pre- and post-amplification tubes, respectively. A representative biological replicate is shown. ( **f** ) Amplification timing of the reactions shown in (e) (mean ± s.e.m, n=3). Differences were n.s. to the reference 200c/75c alone, except for *** (p<0.001, Dunnet’s test) ( **g** ) Effect of yeast tRNA and RNAsecure in end-point colorimetric RT-qLAMP against SARS-CoV-2 RNA. Upper and lower pictures show pre- and post-amplification tubes, respectively. A representative biological replicate is shown. ( **h** ) Amplification timing of the reactions shown in (g) (mean ± s.e.m, n=3). Differences were n.s. (one-way ANOVA). ( **i** ) Effect of yeast RNAs (0.1 μg/μL) and RNAsecure on the *ACTB* RT-qLAMP against pure RNA obtained from mid-nasal swabs. ( **j** ) Relative basal (time 0, t0) fluorescence of the RT-qLAMPs shown in (e-h) (mean ± s.e.m, n=6; +/- RNAsecure were considered together). Differences were significant (p<0.001) when 1 μg/μL was compared to either 0.1 or 0.01 μg/μL. All other comparisons were n.s.

## Description

The emergence and global spread of the Severe Acute Respiratory Syndrome Coronavirus 2 (SARS-CoV-2) have led to an unprecedented public health crisis, emphasizing the urgency to develop rapid and sensitive diagnostic tools. The standard diagnostic method, quantitative Reverse Transcription Polymerase Chain Reaction (RT-qPCR), has been widely utilized for the detection of SARS-CoV-2 due to its high specificity and sensitivity (Dutta et al., 2022) . However, RT-qPCR requires specialized equipment and skilled personnel, limiting its widespread applicability, especially in resource-limited settings.

In recent years, the RT-LAMP technique has gained increasing interest for its ability to rapidly amplify specific RNA sequences under isothermal conditions (Notomi et al., 2000; Yoneyama et al., 2007) . RT-LAMP offers several advantages over RT-qPCR, including simplified instrumentation requirements, rapid turnaround time, and the ability to detect target nucleic acids without the need for complex thermal cycling. As a result, RT-LAMP has gained traction as a promising alternative for SARS-CoV-2 diagnostics (Moore et al., 2021; Choi et al., 2023) .

In the context of nucleic acid-based amplification, the use of additives, including carrier RNA, has been explored in RT-qPCR assays to improve the detection sensitivity (McLeish et al., 2012; Hernandez et al., 2022) . Carrier RNA is often employed as a protective agent to mitigate the detrimental effects of inhibitors, especially RNases, present in complex sample matrices and to stabilize RNA targets during the extraction and amplification processes. Additionally, carrier RNA can act as a surrogate template during the reverse transcription step, further enhancing the overall efficiency of the RT-qPCR reaction.

Inspired by the success of carrier RNA in RT-qPCR, we hypothesize that the inclusion of yeast RNA as an additive in RT-LAMP could similarly facilitate the detection of SARS-CoV-2. Yeast RNA has been utilized as a carrier for various molecular biology applications due to its abundance, stability, and resistance to RNase degradation. However, its potential application in RT-LAMP for viral RNA detection remains largely unexplored. To the best of our knowledge, there is only one previous work in which carrier RNA, namely yeast transference RNA (tRNA), was tested as an additive in colorimetric SARS-CoV-2 RT-LAMP (Wei et al., 2021) . This work showed that ~0.01 μg/μL tRNA (the maximum concentration tested) could improve the detection of the virus in saliva, encouraging further research on this simple way of sample preservation.

In this study, we investigated the impact of yeast RNAs as additives on the performance of RT-LAMP for SARS-CoV-2 detection. Our objective was to establish the conditions in which these additives are compatible with colorimetric RT-LAMP, while preserving the overall efficiency and sensitivity of the reaction. To this aim, we set up a SARS-CoV-2 RT-LAMP that was both quantitative and colorimetric. The colorimetric reaction is based on a pH-sensitive indicator that turns from pink to orange/yellow as the pH drops during the liberation of protons, which accompanies the incorporation of nucleotides into the amplified DNA (Tanner et al., 2015) . To further make this reaction quantitative, we added the fluorometric DNA intercalating dye Syto ^TM^ 9 (Oscorbin et al., 2016) . We also used previously tested primer sets against both the N and E RNA-encoding (N/E) proteins of SARS-CoV-2 (Zhang et al., 2020) . The concentration of the N/E template was set to ~200 copies of naked synthetic viral RNAs per reaction. This template also included ~75 genomic copies of the human DNA. Hereafter, we will refer to this mixed template as 200c/75c. The human template serves for the purpose of signal normalization, as a second RT-LAMP reaction against human *ACTB* is often included in diagnostic tests (Zhang et al., 2020) . Right after the preparation of the reaction tubes, and before the amplification, a photo was taken to check tube colours. In this pre-amplification (pre-amp) condition, the colour should be pink. If the colour changes because of either the sample or any additive, the settings can be considered incompatible with the colorimetric RT-LAMP reaction. After 1h of the RT-LAMP reaction (post-amp), a second photo was taken to see whether the amplification has resulted in a colour shift from pink to orange/yellow, which is a visual indicative of amplification.

We first validated the colorimetric and quantitative RT-LAMP with the synthetic 200c/75c template (Fig 1a,b). We found that the time to positivity (Tp) in the quantitative RT-LAMP was 23.4 ± 0.6 min (mean ± s.e.m, n=13 biological replicates) for N/E, and 16.0 ± 0.6 min (mean ± s.e.m, n=9) for *ACTB* (Fig 1c). Little or no amplification was observed when the 200c/75c template was not present (C-, negative control). Accordingly, the pre-amp colour tubes were pink in all cases, and turned to yellow when amplification was observed by RT-qLAMP (Fig 1b). *ACTB* also amplified efficiently from crude nasal swab samples and total RNA extracted from them (Fig 1d). Since nasal swab samples came from healthy individuals, N/E primers did not amplify.

Next, we included yeast RNAs as additives and performed the colorimetric and quantitative RT-LAMP. Two kinds of yeast RNAs were employed, total RNA (toRNA) and tRNA. Three concentrations were tested: 1, 0.1 and 0.01 μg/μL. In addition, we also determined RT-LAMP performance when combining these carrier RNAs with the RNAase inactivation reagent RNAsecure ^TM^ (RNAsec). The highest toRNA concentration (1 μg/μL) turned the pre-amp colour to yellow, so it is incompatible with the overall purpose of the diagnostic (Fig 1e). In addition, the RT-qLAMP amplification of N/E was highly inhibited at this concentration (Fig 1f). Better results in terms of pre-amp colour were obtained with lower toRNA concentrations (0.1 μg/μL or lower) (Fig 1e,f). The results with tRNA were much better, observing total compatibility with both colorimetric and fluorometric readings even at the highest concentration of 1 μg/μL (Fig 1g,h). In all cases, the combination with RNAsec caused a slight colour shift to orange in pre-amp tubes, advising against this additive in colorimetric RT-LAMP reactions. As for the *ACTB* reaction, neither toRNA nor tRNA gave inhibition (Fig 1i). We must bear in mind, though, that this is a LAMP reaction, rather than a RT-LAMP, as the 75c template is human genomic DNA.

In addition to the RT-LAMP data, we assessed the basal levels of Syto ^TM^ 9 fluorescence after adding the yeast RNAs. This was intended to evaluate the sensitivity of this additive as a normalization spike-in during sample handling and preservation. Our results show that a clear pre-amp fluorescence signal was obtained with the highest concentrations (Fig 1j).

In conclusion, we aimed to evaluate the practical use of yeast RNAs as additives to the SARS-CoV-2 RT-LAMP reaction. We conclude that both the colorimetric and the quantitative reactions tolerate well 1 μg/μL for tRNA, which also provides a sufficient baseline for loading normalization. If the sample normally accounts for 1/10 ^th^ of the RT-LAMP reaction volume, this implies that clinical samples can be treated with up to 10 μg/μL of yeast tRNA as a preservative and normalization spike-in. As for toRNA, the working concentration in the RT-LAMP reaction must be lowered to 0.1 μg/μL (i.e., 1 μg/μL in the clinical sample). The difference between tRNA and toRNA may be due to their relative acidic potential. Both RNAs are commonly provided in unbuffered nuclease-free water, but tRNA is expected to have a more neutral pKa because it is highly folded (Thaplyal and Bevilacqua 2014) , which would make tRNA less capable of changing the pH in the pre-amp condition. By combining the simplicity and speed of RT-LAMP with the potential additive benefits of yeast RNAs, this research contributes to the ongoing efforts in developing rapid and accurate molecular diagnostics for SARS-CoV-2 and lays the groundwork for broader applications of this technology in the field of in-field and on-site diagnosis of this and other infectious diseases.

## Methods

The RT-LAMP reaction was performed in a final volume of 10 μL and set as follows: 5 μL of the WarmStart® Colorimetric LAMP 2X Master Mix with UDG, 1 μL of the corresponding 10X primer set, 1 μL of 0.4 M guanidine hydrochloride, 1 μL of 5 μM SYTO™ 9, 1 μL of the template (or water for the negative control), and 1 μL of the corresponding yeast RNA 10X stock when required. For the RNAsec additive, 0.4 μL were added from a 25X stock. Reactions were levelled to 10 μL by adding the required volume of nuclease-free water.

The template was 200c/75c, fresh crude mid-nasal swab (resuspended in 1 mL nuclease-free water), or total RNA extracted from the crude sample. For RNA extraction, we used the PureLink™ RNA Mini Kit kit according to the manufacturer. The yield after RNA extraction ranged from 2.7 to 4.9 ng/μL.

The primer sets were either N/E or hACTB (Zhang et al., 2020) . Each target consisted of six primers (FIP, BIP, LF, LB, F3, and B3). The N/E set comprised 12 primers since it combines two targets, N and E, to be amplified in a single reaction. A 10X stock was prepared such that the final concentration in the reaction was 0.2 μM for F3 and B3 primers, 1.6 μM for FIP and BIP primers, and 0.4 μM for LF and LB primers.

The RT-qLAMP was run in a QuantStudio™ 5 Real-Time PCR System (Applied Biosystems™) for 1 h. The amplification conditions were set to constant 65 ºC cycles, measuring fluorescence every 30 sec (120 cycles overall). After this, the reaction was slowed down by incubating at 4 ºC. The corresponding Ct was then converted to Tp (time to positivity, in minutes) by dividing by 2. Colorimetric assessment of the RT-LAMP was determined by taking photos over a black background. First, a pre-amp photo was taken right after the reaction setup and before running the reaction in the thermocycler. Then, a second photo was taken after 1 h at 65 ºC (post-amp photo).

Graph plots and statistical calculations were performed with GraphPad Prism 10. One-way ANOVA and post hoc tests for multiple comparisons were performed on biological replicates when required, and the meaningful comparisons are indicated in the plots. For plots in panels (f) and (h), a Dunnet’s post hoc test was used, with no carrier RNA and no RNAsec (just 200c/75c) acting as the reference. A Sidak's post hoc was used in panels (d) and (j). No significance (n.s.) is indicated in the figure legend.

## Reagents

**Table:** 

**Reagent/kit**	**Source**	**Reference**
WarmStart® Colorimetric LAMP 2X Master Mix with UDG, 100 rxns	NEB	M1804
PureLink™ RNA Mini Kit, 50 preps	Invitrogen	12183018A
SARS-CoV-2 Positive Run Control (200c/75c)	Bio-Rad	COV019CE
Yeast tRNA (10 mg/mL), 500 µL	Ambion	AM7119
Yeast total RNA (10 mg/mL), 10 ml	Ambion	AM7118
RNAsecure™ RNase Inactivation Reagent, 1 mL	Ambion	AM7005
SYTO™ 9 Green Fluorescent Nucleic Acid Stain	Invitrogen	S34854
MicroAmp™ Optical 8-Cap Strip	Applied Biosystems	4323032
MicroAmp™ Optical 8-Tube Strip, 0.2 mL	Applied Biosystems	4316567
Sterile, nuclease-free water treated with diethylpyrocarbonate (DEPC), 500 mL	VWR	E476

**Table:** 

**Primer**	**Sequence**
Scov2-E1-F3	TGAGTACGAACTTATGTACTCAT
Scov2-E1-B3	TTCAGATTTTTAACACGAGAGT
Scov2-E1-FIP	ACCACGAAAGCAAGAAAAAGAAGTTCGTTTCGGAAGAGACAG
Scov2-E1-BIP	TTGCTAGTTACACTAGCCATCCTTAGGTTTTACAAGACTCACGT
Scov2-E1-LF	CGCTATTAACTATTAACG
Scov2-E1-LB	GCGCTTCGATTGTGTGCGT
Scov2-N2-F3	ACCAGGAACTAATCAGACAAG
Scov2-N2-B3	GACTTGATCTTTGAAATTTGGATCT
Scov2-N2-FIP	TTCCGAAGAACGCTGAAGCGGAACTGATTACAAACATTGGCC
Scov2-N2-BIP	CGCATTGGCATGGAAGTCACAATTTGATGGCACCTGTGTA
Scov2-N2-LF	GGGGGCAAATTGTGCAATTTG
Scov2-N2-LB	CTTCGGGAACGTGGTTGACC
hACTB-F3	AGTACCCCATCGAGCACG
hACTB-B3	AGCCTGGATAGCAACGTACA
hACTB-FIP	GAGCCACACGCAGCTCATTGTATCACCAACTGGGACGACA
hACTB-BIP	CTGAACCCCAAGGCCAACCGGCTGGGGTGTTGAAGGTC
hACTB-LF	TGTGGTGCCAGATTTTCTCCA
hACTB-LB	CGAGAAGATGACCCAGATCATGT
